# Impact of Thermal, High-Pressure, and Pulsed Electric Field Treatments on the Stability and Antioxidant Activity of Phenolic-Rich Apple Pomace Extracts

**DOI:** 10.3390/molecules29245849

**Published:** 2024-12-11

**Authors:** Diana Plamada, Miriam Arlt, Daniel Güterbock, Robert Sevenich, Clemens Kanzler, Susanne Neugart, Dan C. Vodnar, Helena Kieserling, Sascha Rohn

**Affiliations:** 1 Faculty of Food Science and Technology, University of Agricultural Sciences and Veterinary Medicine, Calea Manastur 3-5, 400372 Cluj-Napoca, Romania; diana.plamada@usamvcluj.ro; 2 Institute of Life Sciences, University of Agricultural Sciences and Veterinary Medicine, Calea Manastur 3-5, 400372 Cluj-Napoca, Romania; 3 Department of Food Chemistry and Analysis, Institute of Food Technology and Food Chemistry, Technische Universität Berlin, Gustav-Meyer-Allee 25, 13355 Berlin, Germanygueterbock@campus.tu-berlin.de (D.G.); clemens.kanzler@tu-berlin.de (C.K.); helena.schestkowa@tu-berlin.de (H.K.); rohn@tu-berlin.de (S.R.); 4 Department of Food Biotechnology and Food Process Engineering, Institute of Food Technology and Food Chemistry, Königin-Luise-Straße 22, 14195 Berlin, Germany; r.sevenich@tu-berlin.de; 5 Department of Crop Sciences, Division of Quality and Sensory of Plant Products, Georg-August-Universität Göttingen, Carl-Sprengel-Weg 1, 37075 Göttingen, Germany; susanne.neugart@uni-goettingen.de

**Keywords:** apple pomace, phenolic compounds, high-pressure treatment, antioxidant activity, stability

## Abstract

Apple pomace, a by-product of apple juice production, is typically discarded as waste. Recent approaches have focused on utilizing apple pomace by extracting beneficial bioactive compounds, such as antioxidant phenolic compounds (PCs). Before these PC-rich extracts can be used in food products, they must undergo food preservation and processing methods. However, the effects of these processes on the composition, stability, and properties of the PC remain insufficiently understood. The present study aimed at investigating the effects of a thermal treatment (TT), a high-pressure thermal treatment (HPTT), and a pulsed electric field treatment (PEF) on the composition and antioxidant activity of PC-rich apple pomace extracts (APEs). Major PCs, including phloridzin, chlorogenic acid, and epicatechin, as well as minor compounds, were identified by liquid chromatography–tandem mass spectrometry (LC-MS/MS) and high-performance thin-layer chromatography (HPTLC). As a stability indicative property, the antioxidant activity was analyzed by a Trolox equivalent antioxidant capacity assay (TEAC), electron paramagnetic resonance spectroscopy, and the Folin–Ciocalteu reagent assay. The results showed that TT at 80 °C increased phloridzin content, likely due to the hydrolysis of bound forms, while higher temperatures and HPTT resulted in a substantial PC conversion. The PEF treatment also caused notable PC conversion, but generally, it had a milder effect compared to TT and HPTT. Hence, low temperatures with and without high pressure and PEF seem to be the most promising treatments for preserving the highest content of major PC in APE. Antioxidant activity varied among the analytical methods, with HPTT showing minor changes despite PC loss compared to the untreated APE. This suggests that other antioxidant compounds in the extracts may contribute to the overall antioxidant activity. This study demonstrates that apple pomace contains valuable PC, highlighting its potential as a health-promoting food additive and the impact of conventional preservation and processing methods on PC stability.

## 1. Introduction

Apples rank among the most widely consumed fruits globally, both in their natural form and as ingredients in various processed products like juice and beverages. The apple juice industry generates millions of tons of apple pomace (AP) as a by-product each year worldwide [1]. Currently, most AP is either used as animal feed or discarded as waste. However, with the increasing emphasis on sustainability, there is growing interest in repurposing AP by extracting valuable functional ingredients, such as phenolic compounds (PCs). These have significant potential for developing new food products with added health benefits [2,3]. Despite this interest, the full potential of AP remains underutilized. One of the main limitations is the inconsistency in AP’s quality, which can vary based on factors such as apple variety, processing conditions, and storage. Additionally, concerns about residues and contaminants, pose challenges to its wider application [4]. However, when trying to be more sustainable in the future, risks need to be diminished and legislation needs to be reconsidered and adapted [5]. To address these risks and facilitate future use, comprehensive chemical characterization and (bio)activity analysis, including antioxidant properties, are essential. Additionally, legislative frameworks will need to be reconsidered and adapted to support the safe and sustainable applications of these by-products in the food industry.

Generally, AP consists of peels, seeds, stems, and a significant proportion of residual pulp. Especially, the peel contains a significant amount of water-soluble vitamins and serves as a source of antioxidant compounds, notably PC [6]. The extraction of PC from AP can be achieved through two categories of techniques: conventional methods, including solid–liquid extraction, liquid–liquid extraction, and maceration, or novel approaches, such as pressurized liquid extraction, microwave-assisted extraction, and ultrasound-assisted extraction. Several solvents and liquid–liquid extraction techniques have been devised specifically for extracting PC. In fact, aqueous–organic solvent mixtures have the capability to extract free PC effectively. The aqueous mixtures of methanol [7], acetone [8], or ethanol [9] are used in the extraction process [10], with methanol/water mixtures yielding the highest amounts of PC [11]. However, for using phenolic-rich extracts in food products, solvents need to be of food-grade quality to ensure safety and compliance. Additionally, robust quality systems and management must be established to guarantee that the extracts meet food-grade standards.

However, before such PC-rich extracts might be used in foods, they need to be subjected to typical food preservation and processing methods for safer and more convenient handling as an ingredient. Consequently, the PC contained in these extracts might be significantly affected, altering their beneficial properties [12]. The most common treatment method for the preservation of foods is a thermal treatment (TT). However, emerging and innovative technologies such as hydrostatic high-pressure thermal treatment (HPTT) and pulsed electric field (PEF) processing are described as gentler. Various conditions can be implemented depending on the specific purpose and targeted product. For instance, pasteurization and sterilization are the most widely utilized methods among the numerous TTs. The common temperatures used for pasteurization typically range between 70 and 80 °C, with the processing times varying from 30 min to just a few seconds, depending on the exact temperature [13]. For sterilization, temperatures are generally between 110 and 121 °C, with times ranging from <10 up to approx. 30 min. For instance, a common sterilization treatment is TT at 121 °C for 7 min, illustrating that at higher temperatures, the required time can be significantly reduced. Moreover, an upcoming preservation technique is the combination of temperature and hydrostatic high-pressure treatment [14]. The U.S. Food and Drug Administration approved this method for the preservation of foods at temperatures lower than 121 °C and 600 MPa [15]. In Europe, food processing using HPTT was assessed by the European Food Safety Authority which stated that this treatment effectively eliminates harmful microorganisms and poses no significant food safety concerns compared to more conventional treatments, and has minimal impact on taste, texture, appearance, or nutritional qualities [16]. During high-pressure processing, pressure changes cause temperature shifts due to reversible adiabatic transformations [17]. The product, placed in a sealed high-pressure chamber, is subjected to controlled pressure, temperature, and time conditions. Compression raises pressure by pumping a medium, while pressure release follows treatment. In adiabatic compression, heat from compression is retained, with the internal energy increase linked to work performed, raising the temperature. Typically, temperature rises by about 3 °C per 100 MPa for water-rich foods [18]. This effect must be considered to prevent over processing during HPP.

Substances such as vitamins, minerals, PCs, and flavor compounds typically exhibit minimal alterations under HPTT conditions [17]. However, the specific interactions and potential changes in the composition of PCs in extracts due to HPTT are not yet fully understood. PEF treatment, in comparison to TT and HPTT, may preserve high-quality foods using lower temperatures and shorter time intervals, retaining their nutritional value [19,20,21,22]. While it is known that PEF can enhance the extraction of PC from a variety of plant matrices, a comprehensive understanding of its effect on PC in phenolic-rich extracts, as well as their resulting properties and stability, is lacking.

Consequently, the aim of the present study was to analyze the PC composition, but primarily the stability and the antioxidant activity, of PC-rich extracts from AP before and after TT, HPTT, and PEF treatments. As a scientific model approach for identifying differences in the different treatments, a more detailed profile of the extracted compounds was gained by the use of aqueous methanol, despite knowing that this might raise concerns when adapting to a large-scale transfer in the future. High-performance thin-layer chromatography (HPTLC) was used to monitor and quantify the major apple PC phloridzin, chlorogenic acid, and epicatechin. Liquid chromatography–tandem mass spectrometry analysis (LC-MS/MS) was employed to identify further major and minor compounds as well as their conversion products. The antioxidant activity of the APE before and after treatment was evaluated using three different assays: the Trolox equivalent antioxidant capacity (TEAC) assay, electron paramagnetic resonance (EPR) spectroscopy, and the Folin–Ciocalteu reagent (FCR) assay.

It was hypothesized that increased temperatures during TT accelerate the degradation of the major PC in APE, resulting in reduced antioxidant activity due to enhanced oxidative and hydrolytic reactions. Moreover, HPTT at increased temperatures results in a more pronounced conversion of phloridzin, chlorogenic acid, and epicatechin compared to TT alone, leading to a more significant reduction in antioxidant activity due to enhanced conversion reactions and interactions under hydrostatic high pressure. It was assumed that PEF treatment induces electrochemical reactions that lead to a milder conversion, which might result in less severe effects on the antioxidant activity.

Hence, this investigation provides insights into the stability of PC-rich AP extracts as health-beneficial and techno-functional food additives, but also highlights the significance of preservation and processing techniques for maintaining the stability and efficacy of these valuable PC.

## 2. Results and Discussion

### 2.1. General Observations on PC-Rich Extracts Before and After the Treatments

**Figure 1:**
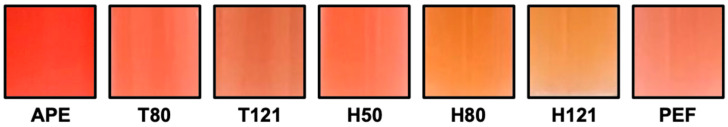
Color of untreated PC-rich apple pomace extract (APE) and treated APE; T80, thermal treatment at 80 °C; T121, thermal treatment at 121 °C; H50, high-pressure thermal treatment at 600 mPa and 50 °C; H80, high-pressure thermal treatment at 600 mPa and 80 °C; H121, high-pressure thermal treatment at 600 mPa and 121 °C; PEF, pulsed electric field treatment.

Figure 1 illustrates the color changes in the APE before and after the different treatments. The untreated APE displayed the most intense reddish color compared to the treated samples. In contrast, all the treatments resulted in lighter shades of red or even yellow and brown. A clear trend was observed: higher temperatures in TT and HPTT lead to more pronounced color changes towards brown or yellow, respectively, while lower temperatures and PEF treatment caused noticeable discoloration. Comparable color changes have been reported in several studies investigating the impact of TT, HPTT, and PEF on other PC-rich (fruit-based) matrices, attributing these changes, among other factors, to the sensitivity of PC to the various treatments [23,24,25].

Generally, PC can undergo various chemical reactions such as oxidation, dehydration, decarboxylation, electrophilic aromatic substitution, and polymerization [26,27,28]. These reactions alter the chemical structure and properties of PC, often resulting in the browning of the extracts and a loss of the initially reddish color, indicating significant changes in the composition of the samples. For HPTT, the combination of thermal impact and increased pressure accelerates chemical reactions, producing yellow or brownish products and consequently leading to more pronounced color changes compared to TT. HPTT is based on the principles of Pascal and Le Chatelier, which state that pressure is uniformly distributed and transferred in all directions, affecting physical properties like solubility, density, viscosity, and equilibrium processes [20,29,30]. This method also influences kinetic events such as reaction rates and acid-base equilibria. Although PEF is generally considered a mild food processing method [31], its impact on chemical reactions is largely unexplored.

### 2.2. Chemical Characterization of PC-Rich Extracts Before and After the Treatments

**Figure 2:**
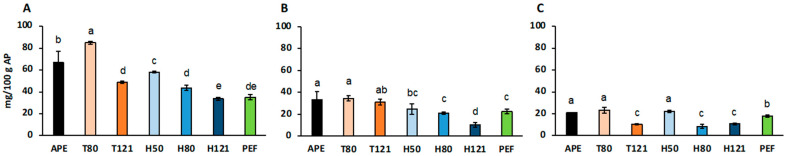
Major phenolic compounds (**A**) phloridzin, (**B**) chlorogenic acid, and (**C**) epicatechin, quantified by HPTLC with untreated PC-rich apple pomace extract (APE) and treated APE; T80, thermal treatment at 80 °C; T121, thermal treatment at 121 °C; H50, high-pressure thermal treatment at 600 mPa and 50 °C; H80, high-pressure thermal treatment at 600 mPa and 80 °C; H121, high-pressure thermal treatment at 600 mPa and 121 °C; PEF, pulsed electric field treatment. Different letters (a, b, c, d, and e) describe the statistically inhomogeneous groups with significant differences (*p* < 0.05).

The quantification of the major PC—phloridzin, chlorogenic acid, and epicatechin—was performed using HPTLC, with the results presented in Figure 2 and Table S1. Additionally, the identification of various minor compounds, including catechin, cyanidin-3-glucoside, quercetin-3-glucoside, quercetin, caffeic acid, and related compounds, was achieved qualitatively by LC-MS/MS (Table 1). The identified PC composition was consistent with those previously reported in apple, apple skin, and pomace [32,33,34]. However, specific degradation products in PC-rich extracts are still rarely characterized and described in the literature. Recently, various treatments have been applied directly to apples or apple juice for the extraction of PC [35,36,37]. To date, these techniques have not been specifically applied to PC-rich extracts alone, and their effects on these extracts remained unexplored.

Phloridzin exhibited the highest content among the PC in the untreated APE and was present at 66.79 mg/100 g AP. Figure 2A shows the significant increase in phloridzin content (84.90 mg/100 g AP) at T80 compared to APE indicating its release from other PC that might be present in APE. The LC-MS/MS analysis revealed the presence of a xyloglucose analog of a phloridzin derivate, identified explicitly as phloretin-2-xylosyl-glucoside. The hydrolysis of the glycosidic bond between glucose and xylose in this compound would yield phloridzin. Other phlorizidin glycosides might also be present. In contrast, all the other treated samples showed a lower content of phloridzin compared to APE, indicating subsequent conversion reactions. For instance, phloridzin is known to form a number of oxidation products: a colorless intermediate, as well as two isomeric forms of a yellow dimer featuring a quinone–quinol structure [38]. These two yellow oxidation products can be potentially responsible for the aforementioned color changes during the treatments (Figure 1).

Generally, a higher temperature was accompanied by a more severe loss of phloridzin (T80 vs. T121 and H50 vs. H80 vs. H121). This temperature dependence has also been described by several other authors [39,40,41]. Remarkably, the conversion of phloridzin was more pronounced under HPTT than under TT. The phloridzin content at T121 was roughly at the same level as at H80, whereas H121 showed a significantly lower phloridzin content than T121. Hence, the additional application of high pressure at the same temperature seems to have a significant impact on the stability of phloridzin. This may be due to increased interactions between phloridzin and other components in the APE under hydrostatic high pressure. Notably, these samples also exhibited the most pronounced yellowish coloration after treatment, indicating the formation of yellow dimers through the oxidation reactions of phloridzin, as previously mentioned (Table 1). High-pressure treatments have been reported to affect phloridzin levels differently depending on the processing conditions and apple varieties. For example, Szczepańska et al. (2021) reported no significant changes in phloridzin levels in apple juice after high-pressure treatment at 300, 450, or 600 MPa for 5 min, suggesting that certain high-pressure conditions might preserve phloridzin [42]. In contrast, Fernández-Jalao et al. (2019) found variable effects depending on the apple variety, with the degradation of phloretin-2-xylosyl-glucoside and phloridzin in Spanish apples after high-pressure treatment at 500 MPa, while Italian varieties exhibited significant increases of approx. 50% [43]. These divergent trends highlight the complexity of phloridzin’s behavior under different high-pressure conditions, likely influenced by factors such as apple variety and specific processing parameters.

The PEF treatment resulted in a nearly 50% reduction in phloridzin content compared to APE. The literature suggests that PEF can facilitate electrochemical reactions that may promote the oxidation of bioactive compounds in food [44]. Given that the PEF-treated samples did not develop a yellowish color, it is likely that the oxidation process stopped at the stage of the colorless intermediate, as previously described. Despite the less pronounced color change compared to H80 and H121, the significant decrease in phloridzin content is particularly concerning, as most studies suggest that PEF treatments do not significantly affect the PC content [45]. This pronounced loss of phloridzin under PEF may also raise concerns about the stability of other easily oxidizable compounds in APE. For example, compounds like catechin and quercetin, which are highly susceptible to oxidative degradation, could similarly experience reductions in concentration following PEF treatment. Furthermore, the absence of yellow discoloration indicates that although oxidation occurred, it did not advance to the later, more colored stages observed under high-pressure treatments, implying that PEF may initiate different oxidation pathways or intensities.

Chlorogenic acid was detected in all the samples with lower contents than phloridzin (Figure 2B vs. Figure 2C). However, for most treatments, the relative loss of chlorogenic acid was less severe, indicating a higher stability compared to phloridzin. The most common reactions of chlorogenic acid responsible for its conversion include dehydration, isomerization, oxidation, formation of lactones, and hydrolysis to quinic acid and to caffeic acid [46,47,48]. In addition, caffeic acid can further be degraded to vinylcatechol or protocatechuic acid. Both, caffeic acid and protocatechuic acid were detected in the samples, as shown in Table 1 and Table S1, with neither compound originally present in the APE, suggesting the degradation of chlorogenic acid. The formation of caffeic acid was most pronounced at the highest temperature of 121 °C in samples T121 and H121, which is in line with the literature suggesting an enhanced conversion of chlorogenic acid above 100 °C [49]. In this context, it should be noted that the TT of hydroxycinnamic acids such as caffeic acid leads to non-enzymatic browning reactions. The colored reaction products would explain the yellowish or slightly brownish color of the strongly heated samples [50]. In the TT samples, only T121 resulted in a significant loss of approximately 10% of chlorogenic acid. Despite this relatively low conversion, the sample exhibited an obvious change in color compared to T80 or untreated APE. This suggests a high relevance of non-enzymatic browning reactions in heat-treated APE as recently pointed out by Feng et al. (2021) [51].

HPTT induced a generally stronger conversion than TT, as already observed for phloridzin. H121 led to a conversion of chlorogenic acid of nearly 70 %, the highest decrease in PC content observed due to treatment of the APE. PEF treatment also caused a significant decrease in the chlorogenic acid content of more than 20 %, but the loss was considerably lower than for phloridzin (nearly 50 %). Schilling et al. (2007) suggested that the effect of PEF on chlorogenic acid content can be reduced by using field strengths of less than 5 kV/cm as applied in the present study [52]. In more concrete terms, the authors observed that field strengths of 3 kV/cm and 1 kV/cm did not show significant changes in chlorogenic acid content, highlighting that milder PEF conditions are effective in preserving chlorogenic acid [52].

The third major PC in AP is epicatechin with an initial content of 21.1 mg/100 g AP in the untreated APE (Figure 2C). This content was lower than the initial amounts of phloridzin and chlorogenic acid. In the literature, epicatechin has been shown to undergo partial conversion reactions at temperatures as low as 50 °C and 80 °C [53]. However, this is not consistent with our findings, which show no significant changes in epicatechin content under similar temperatures with and without high pressure. Specifically, our results indicate that epicatechin remained stable under T80 and H50 treatments, suggesting that the compound may be more resistant to thermal degradation under certain conditions than previously reported. Nevertheless, PEF treatment caused a loss of around 14% of the epicatechin content, whereas all the other treatments (i.e., T121, H80, and H121) showed a more severe impact, reducing its content by 50–60%. As observed for the other major PC, higher temperatures caused generally an increased loss of epicatechin. However, the additional impact of HPTT compared to only TT is less pronounced; while H50 showed a significantly stronger loss of phloridzin and chlorogenic acid than T80, there was no significant difference for epicatechin in the same treatments. Also, H121 and T121 resulted in a comparable decrease in epicatechin content, whereas H121 caused a significantly stronger decrease for the other PC than T121. On a chemical basis, these differences are hard to explain, but are noteworthy nevertheless. So far, it is known from the literature that at lower temperatures (under 80 °C) catechins are prone to epimerization reactions [54,55], whereas temperatures above 80 °C can lead to degradation reactions and at least to non-enzymatic browning reactions that might contribute to the observed color changes [51].

Caffeic acid and quercetin were not detected in the untreated APE (Table S1). Although both PC are commonly reported to be found in apples [56], their content or overall presence can vary depending on apple variety, storage conditions, and extraction method [57]. However, all the treatment methods resulted in the formation of the aglycone quercetin in the samples (Table S1), and caffeic acid was formed after T121, H80, and H121. As mentioned before, caffeic acid is esterified to quinic acid in the form of chlorogenic acid and quercetin is the aglycon of different glycosides. The hydrolysis of ester bonds in chlorogenic acids or glycosidic bonds in quercetin glycosides will release both PCs in their free form. Indeed, a number of quercetin glycosides, namely quercetin-3-rutinoside, quercetin-3-glucoside, quercetin-3-galactoside, quercetin-3-arabinoside, quercetin-3-xyloside, quercetin-3-pentoside, and quercetin-3-rhamnoside, were identified in the APE by LC-MS/MS (Table 1) as potential sources for free quercetin after hydrolysis.

### 2.3. Antioxidant Activity of PC-Rich Extracts Before and After the Treatments

**Figure 3:**
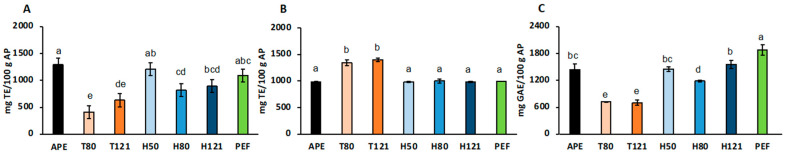
Antioxidant activity analyzed by (**A**) electron paramagnetic resonance (EPR), and (**B**) Trolox equivalent antioxidant capacity (TEAC assay after 120 min) and (**C**) Folin–Ciocalteu reagent (FCR) assay with untreated PC-rich apple pomace extract (APE) and treated APE; T80, thermal treatment at 80 °C; T121, thermal treatment at 121 °C; H50, high-pressure thermal treatment at 600 mPa and 50 °C; H80, high-pressure thermal treatment at 600 mPa and 80 °C; H121, high-pressure thermal treatment at 600 mPa and 121 °C; PEF, pulsed electric field treatment. Different letters (a, b, c, d, and e) describe the statistically inhomogeneous groups with significant differences (*p* < 0.05).

The antioxidant activity of APE before and after TT, HPTT, and PEF was analyzed with EPR spectroscopy, FCR assay, and the TEAC assay (Figure 3). The FCR method is commonly used to determine TPC. However, it is well known that the FCR does not specifically react with PC. Instead, any compound with reducing abilities in a sample may react with the reagent and distort the TPC results. Although PCs are the primary reducing agents in APE [58], it is possible that other reducing compounds were extracted from the AP using methanol or that additional reducing substances were formed during the TT, HPTT, and PEF treatments of APE. Therefore, the results obtained from the FCR method should be interpreted with caution when discussing the TPC. Nevertheless, the FCR results can be considered as antioxidant activity and compared with the EPR and TEAC data. Figure 3 shows the distinct antioxidant activities of the samples after each applied treatment. Notable differences were observed between the EPR results (Figure 3A) and the results of the FCR assay (Figure 3C) on the one hand, and the TEAC method (Figure 3B) on the other hand, which will be discussed in the following section.

The TEAC assay showed a significant increase in the antioxidant activity of approx. 30% for T80 and around 40% for T121. The antioxidant activity of all the other samples remained roughly on the same level (less than 2% difference to APE). EPR and FCR exhibited a decrease in the antioxidant activity for most treatments and generally, both methods reveal the same trends. High temperatures have the most severe effect and reduce the antioxidant activity by around 60% for T80 and 50% for T121, respectively. H50 did not cause a significant change in antioxidant activity, whereas H80 and H121 showed a decrease of 40% and 30%, respectively, with the EPR method. With FCR, H80 had its antioxidant activity decreased by 20% and H121 even showed a slight increase. The PEF treatment resulted in a decrease in antioxidant activity by around 20% as determined by EPR and an increase of 30% as measured by FCR.

**Figure 4:**
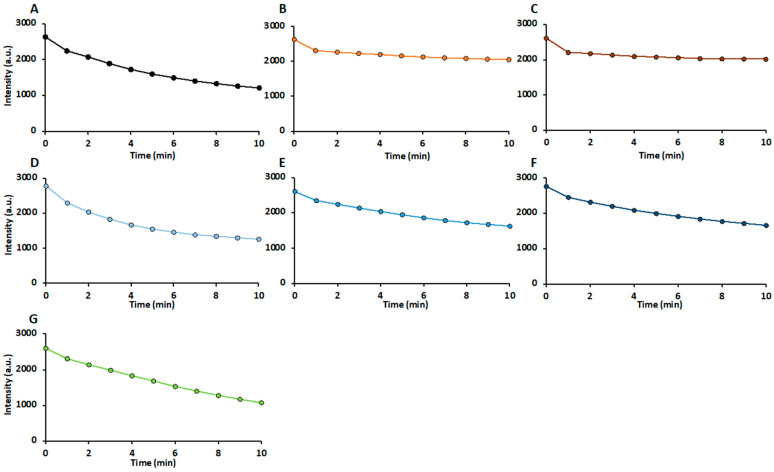
Degradation of Fremy’s salt analyzed by electron paramagnetic resonance (EPR). Samples: (**A**) untreated PC-rich apple pomace extract (APE) and treated APE; (**B**) thermal treatment at 80 °C; (**C**) thermal treatment at 121 °C; (**D**) high-pressure thermal treatment at 600 mPa and 50 °C; (**E**) high-pressure thermal treatment at 600 mPa and 80 °C; (**F**) high-pressure thermal treatment at 600 mPa and 121 °C; (**G**) pulsed electric field treatment.

To highlight the differences between APE and the treated samples in terms of antioxidant composition, the EPR kinetics are shown in Figure 4. The time-dependent radical scavenging displayed here represents the cumulative effect of all individual radical scavenging kinetics of the antioxidants in the samples. The course of the kinetics provides insight into the average reaction rate of the contained antioxidants present. For instance, APE (Figure 4A), H50 (Figure 4D), and PEF (Figure 4G) exhibited comparable overall antioxidant activity, but the reaction rates (slopes of the curves) during the first two minutes differed markedly. PEF showed nearly linear scavenging over 10 min, suggesting the presence of antioxidants that react more slowly. In contrast, H50 (Figure 4D) demonstrated rapid radical degradation in the initial minutes, which slowed over time, indicating the presence of fast-reacting antioxidants that are quickly depleted. The curve of APE (Figure 4A) fell between those of H50 and PEF. Hence, all the samples had different compositions of antioxidants with varying reactivity and stability.

The differences in the antioxidant activity reflect that the radicals or oxidizing agents used in the EPR, TEAC, and FCR methods interact differently with the various antioxidants in the samples. The treatments seem to affect different groups of antioxidants in APE. The content of phloridzin, chlorogenic acid, and epicatechin (Figure 2) decreased or remained unchanged after most treatments. However, changes in the content of these known antioxidants did not directly translate into corresponding changes in the antioxidant activity (Figure 3). Clearly, the total content of the major PC remained significantly lower than the equivalents of Trolox or gallic acid measured by EPR, TEAC, and FCR. Therefore, other free PCs, as well as bound PC and antioxidants from entirely different compound classes, also play a crucial role in the overall antioxidant activity of the samples. Moreover, Asif et al. (2024) recently summarized that the TPC can vary significantly depending on the extraction methods used for PC in apples [59]. This variability in extraction methods may further complicate the understanding of how antioxidant activity is influenced by different phenolic profiles. All these compounds and changes in their respective content are difficult to assess, and further studies are needed to better understand the observed changes in antioxidant activity [60]. At present, the content of major PCs in differently treated APEs cannot be reliably used as a marker for the resulting antioxidant activity. Furthermore, the treatments induce fundamentally different changes in the composition of antioxidants, which can lead to significantly varying results depending on the assessment method used. Therefore, employing more than one method for evaluation seems advisable.

When reconsidering the current results, it is noteworthy that HPTT—the treatment that had the most severe effect on the content of phloridzin, chlorogenic acid, and epicatechin—showed only minor changes in antioxidant activity across all the assays used. Conversely, TT had a strong impact on antioxidant activity, though the direction of this impact varies depending on the method, despite inducing less drastic changes in the content of the three major PC. This suggests that other groups of compounds are significantly affected by TT and HPTT. Overall, considering antioxidant activity across all the methods, the treatments that preferably preserve antioxidant capacity were H50 and PEF, with H121 also being effective to a certain extent.

As mentioned earlier, the FCR method is commonly employed to determine TPC in samples expected to be rich in phenolic compounds, such as apples, apple juice, pomace, or their extracts. However, in the present study, the content of major PCs like phloridzin, chlorogenic acid, and epicatechin did not correlate with the results obtained from the FCR method. While other PCs or their reaction products that were not quantified could account for these discrepancies, this cannot be confirmed based on the current data. However, in the study described by Matias et al. (2024), HPTT did not induce significant changes in TPC of PC-rich smoothies [61], consistent with the findings described by De la Peña-Armada et al. (2022), who reported no significant effect on TPC by Folin–Ciocalteu in apple by-products subjected to different high-pressure conditions [62]. However, these results contrast with other studies, such as those described by Andrés et al. (2016), which reported an increase in PC content after high-pressure treatment in beverages made from other fruits and vegetables [23]. This divergence suggests that the response of PC to HPTT may vary depending on the specific fruit or vegetable matrix being processed, and highlights the complexity of predicting phenolic behavior in different food systems.

In summary, the analysis of the antioxidant activity revealed variations among the TEAC, EPR, and FCR methods. The results indicate that major PC such as phloridzin, chlorogenic acid, and epicatechin do not reliably reflect antioxidant activity on their own, as different treatments affect various antioxidant groups. Therefore, using a combination of assessment methods is essential for a comprehensive evaluation of antioxidant activity.

## 3. Materials and Methods

### 3.1. Materials

Chlorogenic acid (purity ≥ 95%), phloridzin dihydrate (purity 99%), epicatechin (purity ≥ 98%), and quercetin-3-*O*-glucoside (purity ≥ 95%) were purchased from Sigma Aldrich Corp. (St. Louis, MI, USA). Quercetin dihydrate (purity > 95%), caffeic acid (purity ≥ 98%), gallic acid (purity ≥ 98%), and polyethylene glycol 400 were purchased from Fluka AG (Buchs, Switzerland). Phloretin (purity ≥ 98%) and cyanidin-3-*O*-glucoside (purity > 95%) were purchased from Carbosynth Ltd. (Berkshire, UK). Folin–Ciocalteu’s phenol reagent, formic acid (purity > 95%), potassium nitrodisulfonate, (±)-5-hydroxy-2,5,7,8-tetramethyl-chromane-2-carboxylic acid (Trolox) (purity > 97%), and 2,2′-azino-bis(3-ethylobenzothiazoline-6-sulphonic acid)-diamonium salt (ABTS) (purity ≥ 98%) were all purchased from Sigma Aldrich, as well. Catechin (purity ≥ 98%), sodium hydroxide (purity ≥ 99%), ascorbic acid (purity ≥ 99%), citric acid (purity ≥ 99.5%), natrium carbonate (purity ≥ 99.5%), and potassium hydroxide (purity ≥ 85%) were purchased from Carl Roth (Carl Roth GmbH & Co. KG, Karlsruhe, Germany). Methanol (purity ≥ 99.5%), ethyl acetate (purity ≥ 99.5%), diethyl ether (purity ≥ 98%), dichloromethane (purity ≥ 99.5%), hydrochloric acid (37%), toluene (purity ≥ 99.5%), and acetic acid (purity ≥ 99.5%) were purchased from VWR Chemicals GmbH (Darmstadt, Germany). Ethanol (purity ≥ 99.9%) and potassium dihydrogen phosphate (purity ≥ 99.5%) were purchased from Merck KGaA (Darmstadt, Germany), while 2-aminoethyl diphenylborinate (purity ≥ 98%) was purchased from Thermo Fisher Scientific Inc. (Waltham, MA, USA).

### 3.2. Methods

#### 3.2.1. Preparation of Apple Pomace

AP was prepared on a laboratory scale. The endogenously phenol-rich apple (*Malus domestica*) variety ‘Red Delicious’, bought from a local market, was selected as model raw material for the extraction of PC, being renowned for undergoing a fast enzymatic browning [29,63] as an indicator for a high content in PC. To prevent enzymatic browning and consequently, changes in the PC content, a pretreatment was carried out to inactivate the polyphenoloxidase (PPO) activity of the AP [29]. Blanching was carried out by immersing diced apples in water with ascorbic acid and citric acid (1% each, pH 2) at 95 °C for 4 min. The pretreated apples were processed in a commercial, household-scale apple juicer (Bomann^®^ AE 1917 CB professional automatic juicer, C. Bomann GmbH, Kempen, Germany), and further separation of the residual apple juice and the AP was carried out by pressing with the help of a cloth. AP was freeze-dried, and after 24 h, it was ground (IKA MultiDrive control, IKA^®^-Werke GmbH & Co. KG, Staufen, Germany), sieved to obtain a fine powder, and stored at −20 °C until use.

#### 3.2.2. Preparation of Phenolic-Rich Apple Pomace Extracts

Methanol was used as an extraction agent as described by Li et al. (2020) [30]. A total of 500 mg of the AP sample were suspended in 15 mL of chilled methanol/water (80:20, *v*/*v*, 1% formic acid) and then ultra-turraxed (T25 basic, IKA^®^-Werke GmbH & Co. KG, Staufen, Germany) for 15 min at 13,500 rpm. The mixture was centrifuged (Hermle Z320, Hermle Labortechnik GmbH, Wehingen, Germany) for 10 min, at 22 °C and 1398× *g*. The supernatant was separated and the residue obtained was mixed with 40 mL methanol/water (80:20 *v*/*v*, 1% formic acid). The procedure was repeated once. The supernatants were combined and the solvent was removed using a rotary vacuum evaporator at 40 °C. The final dry extract (0.5 g) was dissolved in water (5.0 mL) and stored at −20 °C.

#### 3.2.3. Application of Preservation Treatments

##### Thermal Treatment

The TT was performed at two distinct temperatures, 80 °C for 20 min (T80) and 121 °C for 7 min (T121). The samples in an open round-bottom flask were immersed into an oil bath utilizing a precise thermostat (Polystat cc3, Peter Huber Kältemaschinenbau GmbH, Offenburg, Germany). Subsequently, the samples were promptly cooled on ice. The treated samples were stored at −20 °C until analysis to ensure sample integrity. The experimental procedure was replicated three times and 6 mL of AP extract was utilized for each sample.

##### Hydrostatic High-Pressure Thermal Treatment

The HPTT was performed at three different temperatures 50 °C, 80 °C and 121 °C at a hydrostatic pressure of 600 MPa (referred to as H50, H80, and H121) in a high-pressure multivessel apparatus U111 (Unipress, Warsaw, Poland). For all three temperatures, a preheating time was applied until the desired temperature was reached. Afterward, the samples were kept at 50 °C for 20 min, at 80 °C for 20 min, and at 121 °C for 7 min. Immediately after treatment, the samples were cooled on ice and then stored at −20 °C until analysis. The treatments were applied to 6 mL of each sample and were conducted in triplicate.

##### Pulsed Electric Field Treatment

The PEF treatments were applied using a 5 kW PEF generator HVP 5 (DIL ELEA, Quakenbrück, Germany). The electric pulses of near-rectangular shape were delivered in a batch chamber with two stainless steel parallel electrodes (distance = 2 cm) and a final volume of 50 mL. The PEF treatment had an input voltage of 10.9 kV, a constant pulse width of 18 μs, a constant frequency of 30 Hz, and a constant pulse number of 300 pulses. Prior to the PEF treatment, the solution was heated up to 80 °C in a water bath. The electric field strength (kV/cm), pulse energy, and specific energy input were calculated based on the data provided by the HVP 5 PEF system. The experimental procedure was replicated three times.

#### 3.2.4. Solid Phase Extraction

The solid phase cartridges (SPE Chromabond PA, 6 mL, 500 mg, Macherey-Nagel GmbH & Co. KG, Düren, Germany) were preconditioned with 2 × 5 mL 70% methanol and 2 × 5 mL ultra-pure water. Afterward, 4 mL of untreated APE or of treated samples were loaded onto the preconditioned cartridge and rinsed with 10 mL of ultra-pure water. Finally, the analytes were eluted with 2 × 5 mL methanol/water/acetic acid (90:5:5, *v*/*v*/*v*) into a pear-shaped flask and evaporated to dryness at 40 °C. Ultimately, the residue was taken up in 2 mL HPLC-grade methanol. The samples were stored at −20 °C until used.

#### 3.2.5. Liquid Chromatography–Tandem Mass Spectrometry (LC-MS/MS)

LC-MS/MS was carried out using an Agilent 1290 Infinity II HPLC system consisting of a binary pump and an autosampler (Agilent Technologies Inc., Santa Clara, CA, USA), coupled with a triple quadrupole mass spectrometer (MS-QQQ, Model G6460C, Agilent), equipped with an ESI source. The working conditions of the ESI source in negative ion mode were the following: gas temperature 350 °C, gas flow 13 L/min, nebulizer pressure 60 psi, and capillary voltage 4000 V, and the samples were analyzed in scan mode. The analysis was performed in a gradient elution with a mobile phase of water with 0.1% formic acid (solvent A) and methanol (solvent B) at a constant flow of 0.4 mL/min. The column oven temperature was set at 40 °C. The gradient was as follows: 0.5 min, 5% B; 13 min, 98% B; 16 min, 98% B; 16.50 min, 5% B; and 20 min, 5% B. The injection volume was 5 μL. Data collection and subsequent analysis were conducted using the Agilent LC-ESI QTOF-MS/MS Mass Hunter Qualitative Software B.08.02 (Agilent Technologies Inc., Santa Clara, CA, USA).

#### 3.2.6. High-Performance Thin-Layer Chromatography (HPTLC)

##### Sample Application and Chromatography

Samples were sprayed on HPTLC plates (HPTLC silica gel 60 F254 plates 20 × 10 cm, Merck KGaA, Darmstadt, Germany) using the Automatic TLC Sampler 4 (ATS 4, CAMAG AG, Muttenz, Switzerland). Before analysis, the plates were washed with methanol and activated in an oven (VT-5042-EK Drying oven, Heraeus Deutschland GmbH & Co. KG, Hanau, Germany) at 110 °C for 10 min [64]. The aqueous methanol extracts (20 µL) were sprayed as 6 mm bands with a distance between tracks of 10.6 mm, 8 mm from the bottom, and 15 mm to both sides onto the HPTLC plate. The mobile phase used for the plate development was a mixture of ethyl acetate/toluene/formic acid/methanol (6:6:1.6:0.4, *v*/*v*/*v*/*v*) in a horizontal chamber, accordingly [65]. Before starting the separation, the plate was kept for approx. 15 min in the chamber for solvent saturation. Separation was stopped 1 cm before the solvent front reached the end of the plate. Before further analysis, the plate was air-dried.

**Figure 5:**
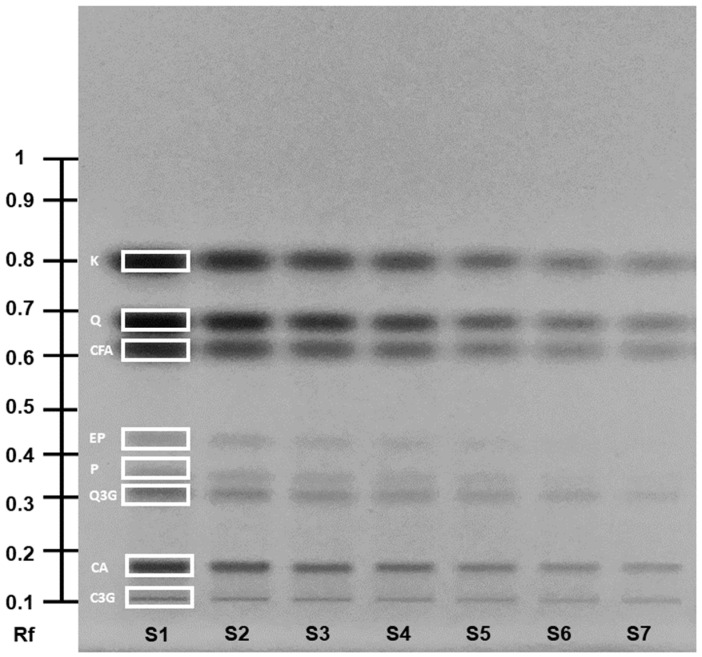
HPTLC plate with the standard calibration of phenolic compounds at 366 nm (mixed standard—3 mg/10 mL—application volume of 25 μL). C3G—cyanidin-3-O-glucoside; CA—chlorogenic acid; Q3G—quercetin-3-O-glucoside; P—phloridzin; EP—epicatechin; CFA—caffeic acid; Q—quercetin; K—kaempferol. S1–S7, standard 1–7: S1—undiluted; S2—dilution 1:1; S3—dilution 1:2; S4—dilution 1:4; S5—dilution 1:6; S6—dilution 1:8; S7—dilution 1:10.

The quantitative evaluation was carried out using a TLC scanner (TLC Scanner 3, CAMAG AG, Muttenz, Switzerland) at 366 nm. A mixed standard was prepared for the calibration: chlorogenic acid 3.5 mg/10 mL, phloridzin 4 mg/10 mL, epicatechin 5.1 mg/10 mL, caffeic acid 2.2 mg/10 mL, quercetin 2.2 mg/10 mL, cyanidin-3-glucoside 2.6 mg/10 mL, quercetin-3-glucoside 2.5 mg/10 mL, catechin 5 mg/10 mL, and phloretin 2.5 mg/10 mL. The calibration curves were generated using six data points between 0.15 and 0.11 mg/10 mL (Figure 5). Standard solutions were prepared in HPLC-grade methanol and analyzed in triplicate. The correlation coefficient (R2) was >0.98 for each regression equation.

##### Post-Chromatographic Derivatization Using Natural Product Reagent

The plate was heated at 110 °C for 3 min before derivatization. For derivatization, two solutions were prepared: (1) 1 g 2-aminoethyl diplenylborinate dissolved in 200 mL ethyl acetate and (2) 10 g polyethylene glycol 400 dissolved in 200 mL dichloromethane. After the plate was heated, it was immersed in the first solution using an automatic immersion device (Chromatogram Immersion Device III, CAMAG AG, Muttenz, Switzerland); after the plate was dried, it was immersed in the second solution. In the end, the derivatized plate was analyzed under UV light at 366 nm. The chromatographic results were analyzed using the HPTLC software winCATS version 1.4.2 (CAMAG AG, Muttenz, Switzerland).

#### 3.2.7. Determination of the Antioxidant Activity by Electron Paramagnetic Resonance Spectroscopy (EPR)

The antioxidant activity of the AP extracts was assessed using EPR. The scavenging of the stabilized radical Fremy’s salt (potassium nitrosodisulfonate) was recorded for 10 min as a function of time. The measurements were carried out in Sorensen’s phosphate buffer at pH 7.4. A 10 mmol/L solution of Fremy’s salt was prepared in the buffer and the working solution was prepared by diluting the stock solution by 1:10 in the buffer. For the calibration, a Trolox stock solution with a concentration of 10 mmol/L was prepared in ethanol. The samples (100 µL) were mixed with 100 µL radical working solution and subjected to EPR measurement (MiniScope MS 100, Magnettech Gesellschaft für Meß- und Steuerungstechnik mbH, Berlin, Germany). The EPR spectra were recorded at a B_0_ field of 3388 G, a sweep of 70 G, a sweep time of 30 s, a modulation amplitude of 1500 mG, a microwave attenuation of 10 dB, and a gain factor of 1 × 10^1^ every minute over a 10 min time period.

The antioxidant activity was calculated at 10 min after radical addition in comparison to a 0.25 mmol/L Trolox solution (Trolox stock diluted with buffer) and expressed as Trolox equivalents (mg TE/100 g dry weight of AP).

#### 3.2.8. Folin–Ciocalteu Reagent Assay

The content of reducing compounds was determined by the Folin–Ciocalteu reagent (FCR) assay [30]. A total of 50 μL of the untreated APE or of the treated samples were mixed with 250 μL 2 M Folin–Ciocalteu reagent and 3 mL of distilled water, vortexed (Vortex Mixer neoLab 7-2020, Heidelberg, Deutschland) for 10 s, and then 1 mL of 15% Na_2_CO_3_ was added. Thereafter, 700 μL distilled water was added to a final volume of 5 mL and vortexed for 10 s. The samples were incubated for 1 h at room temperature in a dark place. After the incubation, the samples were centrifuged at 3146× *g* and the absorbance of the supernatant was determined at 765 nm using a UV/Vis-spectrophotometer (Shimadzu UV-1280, Duisburg, Germany). Calibration was carried out with gallic acid standards in a concentration range from 10 to 125 mg gallic acid/mL and results were expressed as mg GAE/g AP (milligrams of gallic acid equivalents per gram of AP dry weight).

#### 3.2.9. Determination of the Antioxidant Activity by the Trolox Equivalent Antioxidant Capacity (TEAC) Assay

TEAC assay was used to assess the antioxidant activity of APE before and after treatments by measuring the scavenging of the stable ABTS (2,2′-azino-bis(3-ethylobenzothiazoline-6-sulphonic acid)-diamonium salt) radical cation. The results were expressed in Trolox equivalents (mg TE/100 g dry weight of AP). A mixed 1:1 of ABTS solution (10 mmol/L) and a potassium persulfate solution (3.5 mmol/L) was produced and incubated overnight in the dark to obtain the ABTS^•+^ radical cation solution. The ABTS^•+^ solution was diluted 12:100 with phosphate buffer with pH 7.4; the blank value had an extinction of less than 1.4.

The calibration was performed using six Trolox standards with concentrations of 0.01, 0.02, 0.04, 0.06, 0.08, and 0.10 mmol/L. Extracts (500 µL) were mixed with ATBS^•+^ solution (500 µL) and measured after 120 min of incubation at 734 nm in triplicate.

#### 3.2.10. Statistics

All the analyses were carried out in triplicate. The results are shown as mean ± standard deviation (SD). A two-way analysis of variance (ANOVA) and subsequent Fisher’s test were performed using the XLSTAT Statistical software (2018.1.1.62926 version) to analyze significant differences between the samples. In advance, tests for normality (Shapiro–Wilk) and variance homogeneity (Levene’s test) were performed. *p* < 0.05 was selected as the significance level.

## 4. Conclusions

This study demonstrates the impact of various processing methods like thermal treatment (TT), high-pressure thermal treatment (HPTT), and pulsed electric field (PEF) on the phenolic compound (PC) composition and antioxidant activity in apple pomace extract (APE). High temperatures, with or without hydrostatic high pressure, generally lead to the increased conversion of major PC such as phloridzin, chlorogenic acid, and epicatechin. However, these effects are compound-specific; for instance, PEF treatment causes a substantial conversion of phloridzin, moderate conversion of caffeic acid, and lower conversion of epicatechin. Regarding antioxidant activity, the analysis across the TEAC, EPR, and FCR methods revealed variations, as different treatments affected distinct antioxidant groups. Therefore, a combination of these methods is essential for a comprehensive evaluation of antioxidant activity. Despite PC transformations, low-temperature HPTT and PEF treatments are the most effective in preserving both the PC content and antioxidant activity compared to thermal treatments.

This research extends the existing literature concerning the effects of processing complex mixtures, such as apple juice, by utilizing studies of PC-rich extracts. By eliminating matrix effects, this approach facilitates a clearer understanding of how processing influences bioactive compounds like PC. Consequently, this study provides valuable insights into the potential of APE as a source of health-promoting compounds and underscores the importance of selecting appropriate processing techniques, such as PEF and low-temperature HPTT, to optimize these benefits. While this study highlights the critical role of these techniques in preserving the bioactive potential of APE for use as a functional food ingredient or additive, it also highlights the complexity of antioxidant activity, which cannot be fully explained by any single compound. Thus, a comprehensive assessment using multiple analytical methods is recommended to fully evaluate the antioxidant potential of APE.

Moving forward, further research is needed to incorporate model investigations that include additional individual components of complex matrices to assess the influence of various interactions, such as those with proteins or sugars, during processing. Understanding these interactions will provide valuable insights into the bioactive properties of APE and its overall functionality as a health-promoting food additive. Subsequently, refining and scaling these processing techniques for industrial applications will help maximize the potential of APE in food formulations.

## Figures and Tables

**Table 1:** LC-MS/MS qualitative analysis of phenolic compounds in untreated apple pomace extract.

Phenolic Compound	*m*/*z*	APE	T80	T121	H50	H80	H121	PEF
Proanthocyanidin dimer	577	+	+	+	+	+	+	+
Catechin	289	+	+	+	+	+	+	+
Epicatechin	289	+	+	+	+	+	+	+
Chlorogenic acid	353	−	+	+	+	+	+	+
Proanthocyanidin trimer	865	+	+	+	+	+	+	+
Cyanidin-3-glucoside	485	+	+	+	+	+	+	+
Cumaroyl-quinic acid	337	+	+	+	+	+	+	+
Quercetin-3-rutinoside	609	+	−	−	−	−	−	−
Quercetin-3-glucoside	463	−	+	+	+	+	+	+
Quercetin-3-galactoside	463	+	+	+	+	+	+	+
Quercetin-3-xyloside	433	+	+	+	+	+	−	+
Quercetin-3-arabinoside	433	+	+	+	+	+	−	+
Quercetin-3-pentoside	433	+	+	+	+	+	−	+
Phloretin-2-xylosyl-glucoside	433	+	+	+	+	+	+	−
Phloretin-2-glucoside	471	+	−	+	+	+	+	+
Quercetin-3-rhamnoside	447	+	+	+	+	+	+	+
Quercetin	301	−	+	+	+	+	+	+
Kaempferol	285	−	+	+	+	−	+	−
Dicaffeoylquinic acid	515	+	+	+	+	+	+	+
Phloridzin oxidation product	467	+	+	+	+	−	+	−
Protocatechuic aldehyde	137	−	−	+	−	+	+	+

## Data Availability

The original contributions presented in this study are included in the article/Supplementary Material. Further inquiries can be directed to the corresponding author.

## Supplementary Materials

The following supporting information can be downloaded at: https://www.mdpi.com/article/10.3390/molecules29245849/s1, Table S1: Quantified phenolic compounds in apple pomace extract by HPTLC.
