# Supplementary material for: Impact of Thermal, High-Pressure, and Pulsed Electric Field Treatments on the Stability and Antioxidant Activity of Phenolic-Rich Apple Pomace Extracts
Source: Molecules. 2024 Dec 11;29(24):5849. doi: 10.3390/molecules29245849 (PMC11678205; doi:10.3390/molecules29245849)
Supplement: Supplementary file 1 [file molecules-29-05849-s001.zip › molecules-3293605-supplementary.pdf]

Supporting information

Impact of Thermal, High-Pressure, and Pulsed Electric Field Treatments on the Stability and Antioxidant Activity of Phenolic-Rich Apple Pomace Extracts

Diana Plamada [1,2,3], Miriam Arlt [3], Daniel Güterbock [3], Robert Sevenich [4], Clemens Kanzler [3], Susanne Neugart [5], Dan C. Vodnar [1,2*], Helena Kieserling [3] and Sascha Rohn [3]

1 Faculty of Food Science and Technology, University of Agricultural Sciences and Veterinary Medicine, Calea Manastur 3-5, 400372 Cluj-Napoca, Romania; diana.plamada@usamvcluj.ro, dan.vodnar@usamvcluj.ro
2 Institute of Life Sciences, University of Agricultural Sciences and Veterinary Medicine, Calea Manastur 3-5, 400372 Cluj-Napoca, Romania; diana.plamada@usamvcluj.ro, dan.vodnar@usamvcluj.ro
3 Department of Food Chemistry and Analysis, Institute of Food Technology and Food Chemistry, Technische Universität Berlin, Gustav-Meyer-Allee 25, 13355 Berlin, Germany; m.arlt@tu-berlin.de; gueterbock@campus.tu-berlin.de, clemens.kanzler@tu-berlin.de; helena.schestkowa@tu-berlin.de; rohn@tu-berlin.de
4 Department of Food Biotechnology and Food Process Engineering, Institute of Food Technology and Food Chemistry, Königin-Luise -Straße 22, 14195 Berlin, Germany; r.sevenich@tu-berlin.de
5 Department of Crop Sciences, Division of Quality and Sensory of Plant Products, Georg-August-Universität Göttingen, Carl-Sprengel-Weg 1, 37075 Göttingen, Germany; susanne.neugart@uni-goettingen.de
* Correspondence: dan.vodnar@usamvcluj.ro; Tel.: +40747341881

Table S1. Quantified phenolic compounds in apple pomace extract by HPTLC

Phenolic compound (mg/100 g AP)	Major compounds			Minor compounds	
	**Phloridzin**	**Chlorogenic acid**	**Epicatechin**	**Caffeic acid**	**Quercetin**
**APE**	66.79 ±1.02[b]	33.50 ± 0.10[a]	21.09 ±0.55[a,b]	n.d.*	n.d.
**T80**	84.90 ± 1.64[a]	34.51 ± 0.16[a]	23.47 ± 0.71[a]	n.d.	3.79 ± 0.20[a]
**T121**	48.65 ± 0.92[c,d]	30.91 ± 0.97[a,b]	10.24 ± 0.32[c]	7.66 ± 0.28[b]	n.d.
**H50**	57.96 ± 1.11[b,c]	24.84 ± 0.14 [b,c]	22.18 ± 0.48[a]	n.d.	n.d.
**H80**	43.46 ± 0.68[d,e]	21.11 ± 0.12[c]	8.25 ± 0.11[c]	0.30 ± 0.09[c]	n.d.
**H121**	33.64 ± 0.09[e]	10.35 ± 0.05[d]	10.69 ± 0.19[c]	8.97 ± 0.15[a]	1.41 ± 0.04[b]
**PEF**	35.06 ± 0.43[c,d]	22.57 ± 1.03[c]	18.01 ± 0.29[b]	n.d.	n.d.
**m/z**	471	353	289	179	301
**RT**	33.19	10.25	12.66	–	–
**Classes**	Dihydrochalcones	Hydroxycinnamic acids	Catechins	Hydroxycinnamic acids	Flavonols

*n.d., not detected; data are mean values of three triplicate determinations. The different letters (a,b,c, and d) describ[illegible] the statistically homogeneous groups without significant differences ($p < 0.05$)
